# Bibliometric Overview on T-Cell Intracellular Antigens and Their Pathological Implications

**DOI:** 10.3390/biology13030195

**Published:** 2024-03-19

**Authors:** Beatriz Ramos-Velasco, Rocío Naranjo, José M. Izquierdo

**Affiliations:** Centro de Biología Molecular Severo Ochoa, Consejo Superior de Investigaciones Científicas, Universidad Autónoma de Madrid (CSIC/UAM), C/Nicolás Cabrera 1, Cantoblanco, 28049 Madrid, Spain; bramos@cbm.csic.es (B.R.-V.); rnaranjo@cbm.csic.es (R.N.)

**Keywords:** TIA1/TIA-1, TIAL1/TIAR, bibliometric analysis, ontology network, human pathologies

## Abstract

**Simple Summary:**

T-cell intracellular antigen 1 (TIA1/TIA-1) and its paralog TIA1-related/like protein (TIAL1/TIAR) have been implicated in the regulation and/or modulation of gene expression through aspects of RNA metabolism, such as (1) transcription, through their interaction with DNA and RNA polymerase II; (2) alternative processing of messenger pre-mRNAs, through selection of canonical and atypical 5′ and 3′ splice sites; (3) localization, stability and/or translation of eukaryotic messenger RNAs, through interaction with 5′ and 3′ untranslatable regions; and (4) control of biological programs fundamental to cell viability (i.e., development, inflammation, proliferation/differentiation, apoptosis, autophagy, responses to stress or viral infections). It is therefore essential to understand the role played by these multifunctional regulators in the establishment and adaptation of the diversity of the transcriptome, translatome, proteome and interactome, which represents a key step in understanding the differences in functional heterogeneity between cells, tissues and organisms with similar genetic complexities. In other words, it is necessary to highlight the importance of identifying the early and late cellular processes and molecular mechanisms where TIA proteins participate and how they contribute to maintain homeostasis, preventing the development and/or progression of deleterious phenotypes. Knowledge of the regulatory dynamics associated with these intracellular antigens will serve as a basis for the identification of future therapeutic strategies.

**Abstract:**

T-cell intracellular antigen 1 (TIA1) and TIA1-like/related protein (TIAL1/TIAR) are two members of the classical family of RNA binding proteins. Through their selective interactions with distinct RNAs and proteins, these multifunctional regulators are involved in chromatin remodeling, RNA splicing and processing and translation regulation, linking them to a wide range of diseases including neuronal disorders, cancer and other pathologies. From their discovery to the present day, many studies have focused on the behavior of these proteins in order to understand their impact on molecular and cellular processes and to understand their relationship to human pathologies. The volume of research on these proteins in various fields, including molecular biology, biochemistry, cell biology, immunology and cancer, has steadily increased, indicating a growing interest in these gene expression regulators among researchers. This information can be used to know the most productive institutions working in the field, understand the focus of research, identify key areas of involvement, delve deeper into their relationship and impact on different diseases, and to establish the level of study associated with them.

## 1. Introduction

TIA1/TIA-1 is an RNA binding protein (RBP) originally discovered in T lymphocytes [1,2], and later found to be expressed in various tissues [3]. Since its discovery by Paul Anderson in 1991 [1], TIA1 has been implicated in myriad cellular and pathological processes due to its widespread cellular location and interaction with various RNAs [4].

Another important RBP is its paralog TIAR/TIAL1, which was identified a year later by the same team. TIAR is involved in the regulation of many aspects of gene expression, either independently or in combination with TIA1 [5]. Although both proteins are widely expressed in human tissues, their distribution differs. TIA1 is highly expressed in the kidney and gonads, with significant expression in the small intestine, lung, skeletal muscle and pancreas [4], whereas TIAR is predominantly expressed in the brain and skeletal muscle, with lower expression in the heart, kidney and lung [6].

Both proteins play important roles in the post-transcriptional regulation of gene expression and are involved in various cellular processes [7,8,9]. However, they also have distinct functions and roles within the cell, such as RNA binding properties [10,11,12], regulation of alternative splicing [7,13,14,15], formation of stress granules [16,17,18,19], involvement in immune responses, apoptosis [20,21,22,23], cell cycle/growth regulation [9,23,24,25] and cellular differentiation/proliferation [26,27]. They often co-operate in signaling processes, but their specific roles can vary depending on the cellular context and RNA/DNA targets.

Given their critical role in post-transcriptional gene regulation, the dysregulation of their functions has been implicated in various diseases and pathological conditions, including neurodegenerative diseases and neurological disorders [28,29,30,31,32,33], various cancers [23,34,35,36,37], autoimmune diseases [38,39], viral infection and/or replication [40,41,42,43,44] and inflammatory disorders [45,46,47,48]. Much attention has therefore been paid to better understanding their involvement in molecular and cellular events and processes, and their relationship with human physio(patho)logy.

A bibliometric review of TIA1 and TIAR could provide valuable insights into the research landscape of these important proteins, which may help to identify research gaps, new areas of interest, potential research partners/possible collaborations between researchers and areas where additional research may be needed. Research on TIA1 and TIAR proteins can be found in various fields, including molecular biology, biochemistry, cell biology and immunology. Notably, the volume of research on these proteins has been steadily increasing, indicating a growing interest in the field.

## 2. Features of Analyzed Publications

We analyzed the scientific literature on TIA1 and TIAR using the Bibliometrix tool [49]. Although Bibliometrix is programmed in the R language, we used biblioshiny, a web-based app included in the bibliometrix package, which allows non-coders to use Bibliometrix. To evaluate the development of research on TIA1 and TIAR since their discovery, we conducted a search in the PubMed database using the advanced search terms *((TIA1[Title/Abstract]) OR (TIA-1[Title/Abstract])) OR (TIA1[Text Word])) OR (TIA-1[Text Word])) NOT (ischemic)*, and *((TIAR[Title/Abstract]) OR (TIAL1[Title/Abstract])) OR (TIAR[Text Word])) OR (TIAL1[Text Word]))*. The exclusion of the term “ischemia” was necessary because of the use of the abbreviation TIA for “transient ischemic attack”.

We gathered the publications related to TIA1 and TIAR and imported the data into Bibliometrix by creating a data frame containing the relevant results in the PubMed format. We started biblioshiny digiting: *bibliometrix::biblioshiny* and submitted the data to perform various analyses to prepare several visualization tools, such as publication graphs, term maps or the most relevant authors in this field, to explore and interpret the data. We also used VOS viewer, a software tool for constructing and showing bibliometric co-occurrence networks.

**Figure 1:**
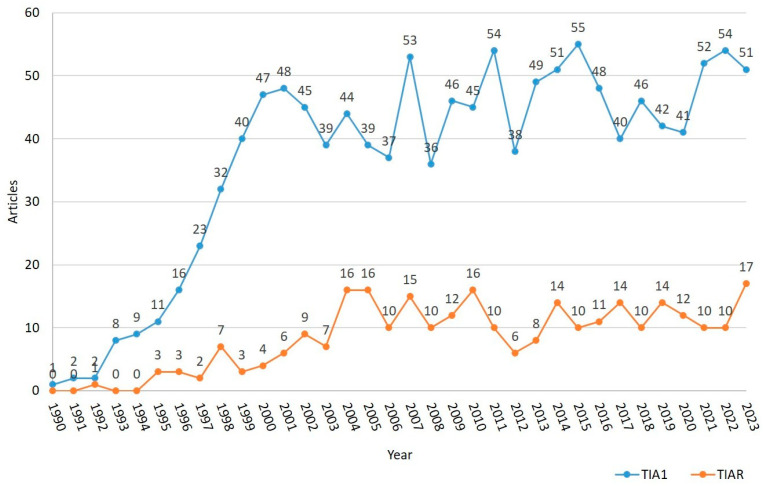
Comparative number of publications on TIA1/TIA-1 and TIAR/TIAL1 per year, according to PubMed (https://pubmed.ncbi.nlm.nih.gov, accessed on 12 March 2024).

A total of 1530 publications were included in the analysis. We collected information from 1244 articles on TIA1/TIA-1 published between 1990 and 2023, and 286 scientific bibliographies on TIAR/TIAL1 published between 1992 and 2023. Since their discovery [1,5], the number of publications on TIA1 has increased considerably until 2002 and on TIAR until 2005, when they reached 48 and 16 publications, respectively. From these dates onwards, the scientific production on TIA1 has fluctuated over time, with sporadic increases in the number of publications, reaching a maximum of 55 articles in 2015. In the case of TIAR, the number of publications was more uniform, ranging from 10 to 17 publications per year, with a minimum of 6 in 2012 (Figure 1). The average growth rate from 1990 to 2023 was approximately 20% (TIA1 22%; TIAR 23%).

**Figure 2:**
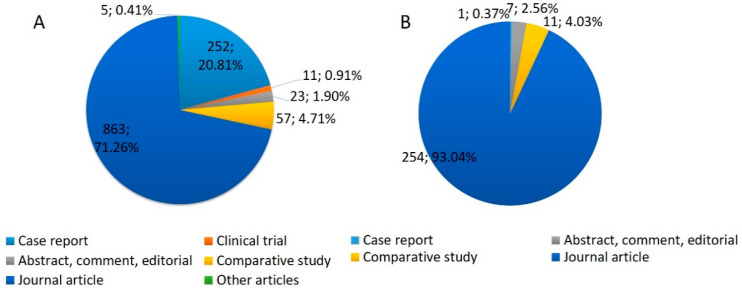
Distribution of publication types on TIA1/TIA-1 (**A**) and TIAR/TIAL1 (**B**) since 1.990 and 1.992, respectively.

We classified all publications into the following types: case reports, clinical trials, abstracts/comments, comparative studies, journal articles and other (Figure 2). In both cases, the “journal articles” group had the highest scientific production of all publications, with 71.26% and 93.04% for TIA1 and TIAR, respectively.

**Figure 3:**
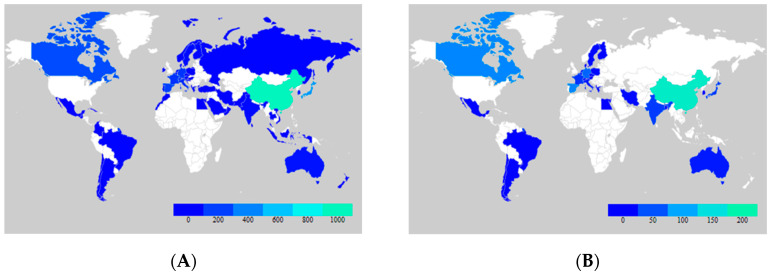
World-wide scientific production on TIA1/TIA-1 (**A**) and TIAR/TIAL1 (**B**).

We also classified the number of publications by country/region (Figure 3), which showed that most publications on TIA1 and TIAR were concentrated in the USA, China, Spain and Germany.

On the other hand, the top 10 journals for publications on TIA1 and TIAR are listed in Table 1, respectively.

## 3. TIA1- and TIAR-Related Networking Analysis

**Figure 4:**
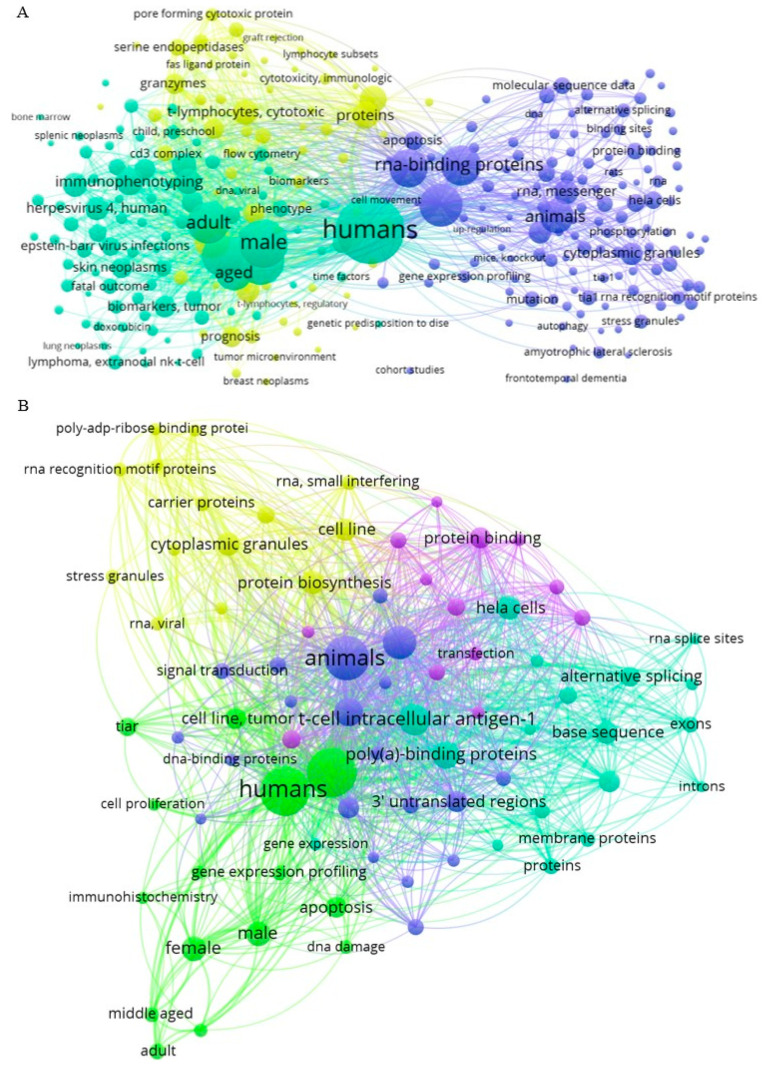
Visualizations of the co-occurrence network of the keywords and terms used in the publications related to TIA1/TIA-1 (**A**) and TIAR/TIAL1 (**B**) from PubMed bibliographic data files [68].

Individual comparison of the co-occurrence networks formed by the TIA1 and TIAR datasets revealed unique keyword clusters for each protein as well as differences between them. The cluster related to TIA1 (Figure 4A) was intricately linked to a diverse set of keywords, emphasizing its critical role in various aspects of molecular and cellular biology, including immune function and cellular cytotoxicity, as well as various aspects of oncology.

In the network related to TIA1, terms such as “humans” (linked also to “female” and “male” terms) and “animals” (such as “mice”) were found. In this case, the “humans” keyword was also connected to certain age ranges/periods/stages including “adult”, “middle aged”, “aged”, “80 and over”, underlining the importance of TIA1 in these groups. We also found keywords related to younger ages, such as “young adult”, “adolescent” or “child, preschool”, albeit more distant from the main node.

TIA1 was prominently linked to keywords such as “RNA-binding proteins”, “gene expression regulation”, “apoptosis” and “alternative splicing”. Within this network, TIA1 is recognized for its influence on “mRNA stability”, particularly through its interactions with RBPs including “Poly(A)-Binding Proteins”. Its involvement in “gene expression regulation”, “gene expression profiling” and “gene expression” and “microRNAs” further highlighted its significance in post-transcriptional control. The presence of TIA1 was also evident in the context of “stress granules”, which are crucial components of cellular responses to physiological stressors. This network underscores the multifaceted role of TIA1 in RNA processing and stress responses, and its potential impact on several diseases, including neurodegenerative disorders such as “Amyotrophic Lateral Sclerosis” (ALS) [50].

Notably, TIA1 emerged as a key player in the understanding of “lymphoma”, particularly “T-cell lymphomas” and “large B-cell lymphomas”. In this line, the use of “immunohistochemistry”, “immunophenotyping” and “flow cytometry” techniques allows researchers to study the expression and immunoreactivity of TIA1 in tumor samples, as evidenced by the keywords “biopsy”, “skin neoplasms” and “lymphomas” in general, shedding light on its oncogenic function and its potential as a diagnostic biomarker for neoplasm staging [51,52,53]. Additionally, the association of TIA1 with “Epstein–Barr Virus” infections underscores its role in the immune response against viral pathogens, which is also related to certain intestinal T-cell lymphomas [54]. The network also addressed “survival analysis”, “treatment outcomes” and “retrospective studies”, highlighting the impact of TIA1 in clinical research and patient prognosis.

Finally, the TIA1 co-occurrence network revealed the close association between TIA1 and the cytotoxic activities of “T-lymphocytes”, particularly “CD8-positive T-lymphocytes” and “CD4-positive T-lymphocytes”, which play a central role in immune response and in oncology [55,56]. Within this network, TIA1 was linked to proteins involved in “cytotoxicity”, including “granzymes” and “perforin”, highlighting its importance in the regulation of immune effector mechanisms [57]. The presence of terms such as “membrane proteins”, “CD8 antigens” and “membrane glycoproteins” also underscores the involvement of TIA1 in surface interactions and signaling processes of immune cells.

The co-occurrence network cluster analysis of TIAR (Figure 4B) revealed connections in the field of RNA biology and cellular regulation. Similar to the TIA1 cluster, several major categories were found for TIAR, including “humans” (linked also with “female” and “male” terms) and “animals” (such as “mice” and “rats”), and “RNA-binding proteins”.

This network included investigations into the involvement of TIAR in “alternative splicing” events, where it influences the inclusion or exclusion of “exons” in mRNA transcripts [11,58], and also its role in “apoptosis” and in the formation of “cytoplasmic granules”, including “stress granules”, during “physiological stress” [59,60,61]. TIAR interactions with RNA, including “mRNA binding” and association with “3′ untranslated regions”, were a central theme of this cluster and revealed the contribution of TIAR to “RNA stability”, also influencing “stress granules”, “gene expression” control and post-transcriptional regulation of “gene expression” [62,63,64,65]. Accordingly, TIAR plays a significant role in “protein synthesis” and is involved in the regulation of “mRNA stability”, often interacting with “Poly(A)-Binding Proteins” to modulate the fate of “messenger RNAs” (mRNAs). Additionally, the network highlighted the links between TIAR and other RBPs including “TIA1” and “ELAVL1”, suggesting collaborative roles in post-transcriptional regulation and underscoring the importance of these RBPs in shaping the cellular landscape of RNA metabolism and stability, immunity and “protein synthesis” [66,67]. Further, experimental techniques such as “Western blotting” and “molecular sequence data” analyses were also widely used in this case.

**Figure 5:**
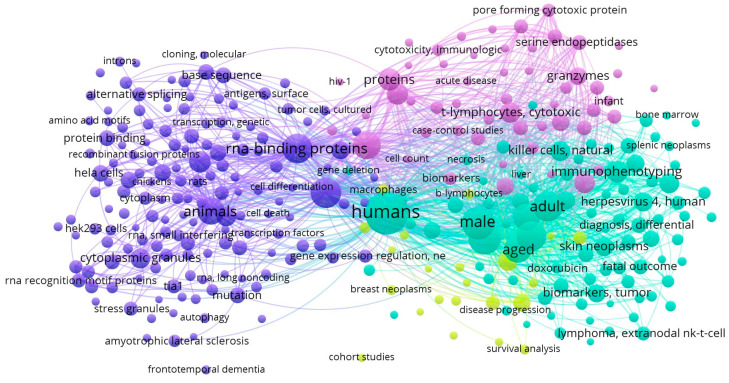
Map of the co-occurrence network of the keywords and terms used in publications related to TIA1/TIA-1 and TIAR/TIAL1 based on the bibliographic data [68].

Comparing the co-occurrence networks of keywords and terms related to TIA1 and TIAR revealed the most common keywords and terms in the publications and how they are related to each other (Figure 5). This was useful to identify patterns and trends in the research on TIA1 and TIAR and to highlight new areas of research and/or potential collaborations. In terms of similarity, the TIA1 and TIAR networks shared common keywords related to “RNA-binding proteins”, “Poly(A)-Binding Proteins”, RNA regulation, “gene expression” regulation, “alternative splicing”, “messenger RNAs”, “cytoplasmic granules”, “cytotoxicity” and “apoptosis”, indicating a common focus on RNA regulation and processing in the study of these two proteins and their involvement in post-transcriptional gene regulation and stress response mechanisms.

On the other hand, it was noteworthy that the most frequently repeated words in both networks are related to human subjects and age groups (“male”, “female”, “adult”, “aged”). In turn, these terms are associated with retrospective studies and to diagnostic techniques such as “in situ hybridization” and “immunohistochemistry”. These techniques are methods used to study gene expression and to investigate the properties and subcellular localization of RBPs and, in this case, the functional roles of TIA1 and TIAR [10,69]. This shared use of the experimental methods highlights the convergence of research strategies employed to determine the role of TIA1 and TIAR in RNA regulation and cellular processes, as a common research toolkit. Furthermore, these common features indicate that both proteins are of interest in clinical and diagnostic studies, particularly in cancer-related research, as suggested by the presence of the terms “tumor”, “skin neoplasms”, “lymphoma” and “treatment outcome”. In addition, keywords related to cytotoxicity, T-lymphocytes, viral infections (including Epstein–Barr Virus/herpesvirus 4) and immune response mechanisms are shared in the cluster, highlighting their importance in immune-related functions and surveillance and viral pathways. Collectively, these findings demonstrate that both proteins contribute to the regulation of cytotoxic T-lymphocytes, which are critical for immune responses against viruses and cancers [4,6,36], including lymphoma.

## 4. TIA1- and TIAR-Associated Molecular Functions and Biological Processes

To collect bibliometric information about the main molecular functions, biological processes and human diseases that our genes of interest are involved in, we utilized the Enrichr suite of gene-set enrichment analysis tools, and we then contrasted the results with the PubMed database publications. A dual strategy was followed for the Gene Ontology (GO)-related Enrichr analysis. First, TIA1 and TIAL1 genes were independently introduced to identify the related biological processes, functions and pathologies. As TIA1 and TIAR are RBPs, their effects may impact the pathways regulated by their RNA targets. For this reason, the sets of genes obtained with the TIA1 and TIAR in vivo crosslinking and immunoprecipitation (iCLIP) analysis [14] were introduced to analyze the indirect effects of TIA1 and TIAL1 on a wider range of biological processes and pathologies.

Gene ontology (GO) is a functional term that considers different aspects of how gene functions can be described. We focused on studies describing the GO molecular functions and biological processes of TIA1 and TIAR; specifically, their involvement in these processes using mRNAs sets of interactions with both TIA1 and TIAR in HeLa cells based on TIA1 and TIAR iCLIP analysis [14]. This analysis allows us to gauge the extent to which these proteins are involved in the control of biological processes.

**Figure 6:**
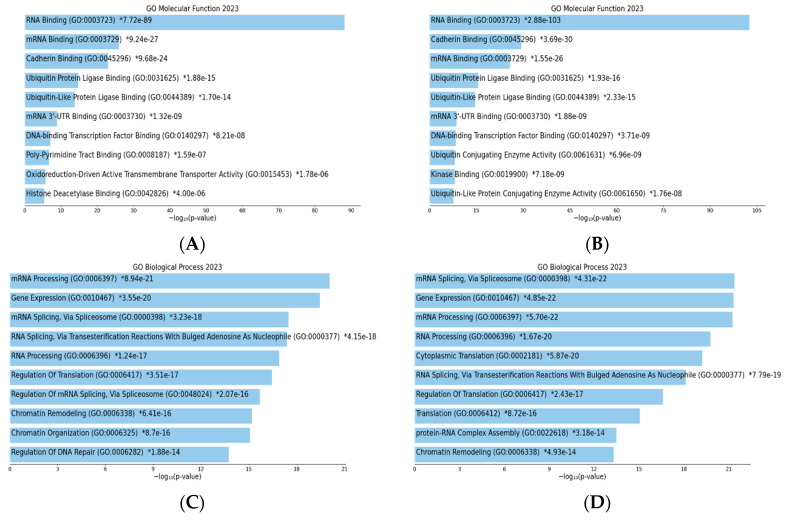
Bar charts of top enriched terms from the GO_Molecular_Function_2023 gene set library for TIA1/TIA-1 (**A**) and TIAR/TIAL1 (**B**), and top enriched terms from the GO_Biological_Process_2023 gene set library for TIA1/TIA-1 (**C**) and TIAR/TIAL1 (**D**) from iCLIP analysis in HeLa cells. The top 10 enriched terms for the input gene set are displayed based on the −log10 (*p*-value), with the *p*-value shown next to each term. The term at the top has the most significant overlap with the input query gene set [70,71,72].

Although the results of the GO molecular function analysis (Figure 6A,B) were almost identical, a review of the biological processes (Figure 6C,D) regulated by the two proteins revealed some differences. This suggests that, despite similar functions such as binding to RNA, cadherins and transcription factors, the different targets of each protein reflect their involvement in specific biological processes. For example, mRNA processing was the top function of TIA1, whereas it occupied third place among the functions of TIAR, with TIAR being more involved in the regulation of splicing by the spliceosome. Similarly, TIA1 was more involved than TIAR in chromatin reorganization.

## 5. Implications of TIA1 and TIAR in Human Pathologies

Previous studies in the literature have reported the involvement of TIA1 in neuropathologies including ALS, tauopathies, spinal muscular atrophy (SMA), stress-related psychiatric disorders, Huntington’s disease (HD), Welander distal myopathy (WDM), tumorigenesis, diabetes and lipid metabolism [4].

In the case of TIAR, its involvement has been reported in inflammation, embryogenesis, carcinogenesis and neurodegenerative diseases such as neurofibromatosis type I, axon regeneration and Alzheimer’s disease [6].

Thus, several diseases/disorders are associated with TIA1 and TIAR expression/dysfunction, including tumorigenesis, acute inflammatory responses, autoimmunity, infectious diseases and neurological disorders, which we investigated and compared with the results obtained from the DisGeNET gene set library and the Jensen list.

As in the previous section, we analyzed the possible involvement of TIA1 and TIAR in different diseases by searching for the set of genes with which they interact.

**Figure 7:**
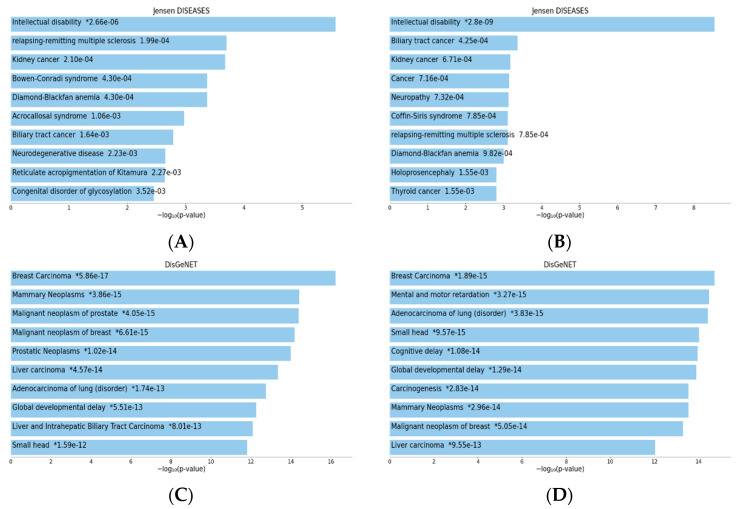
Bar charts of top enriched terms from the Jensen_Diseases gene set library for TIA1/TIA-1 (**A**) and TIAR/TIAL1 (**B**), and top enriched terms from the DisGeNET gene set library for TIA1/TIA-1 (**C**) and TIAR/TIAL1 (**D**). The top 10 enriched terms for the input gene set are displayed based on the −log10 (*p*-value), with the actual *p*-value shown next to each term. The terms at the top have the most significant overlap with the input query gene set [69,70,71].

In the Jensen DISEASES analysis (Figure 7A,B), both proteins appear to be highly involved in intellectual disability. However, TIA1 was ranked second in relapsing-remitting multiple sclerosis, whereas TIAR was ranked seventh. TIAR was more involved in several types of cancer, whereas TIA1, although also involved in kidney cancer, had more varied effects. Both are also involved in neuronal disorders, such as neurodegenerative diseases (TIA1) and neuropathy and holoprosencephaly (TIAR).

Regarding the results of the DisGeNET analysis (Figure 7C,D), cancer was the most relevant for both proteins, especially breast cancer. Both proteins, although with different probabilities, are also involved in small head and global developmental delay, which coincides with the involvement in neuronal disorders seen in the Jensen analysis.

## 6. Perspectives

Since its discovery in 1991 by Anderson et al. [1,5], research on TIA1 (and its paralog TIAR) gradually increased and remained at a stable level for the next 20 years, likely due to its study by established research groups. Research on TIA1 is much more widespread at the geographical level than that of TIAR, perhaps due to its greater relevance to certain biological processes.

The co-occurrence representations provide an insight into the different research directions and interests for TIA1 and TIAR, revealing common and divergent research themes, key areas of investigation, and some areas for further research. They serve as a useful visual representation to identify major keywords, research trends and links in the literature surrounding these proteins. These observations also illustrate the multifaceted nature of TIA1 and TIAR in immune response, cancer biology/oncology, viral infections and RNA biology, making them key players in understanding RNA dynamics and cellular stress responses. Accordingly, their similarities and differences provide a basis for understanding their common and distinct contributions to various biological processes and diseases and/or for exploring potential synergistic interactions and therapeutic implications involving both proteins.

In this way, TIA1 and TIAR RBPs were first described as post-transcriptional regulators of pre-mRNA splicing events, as well as repressors of translation and/or (m)RNA turnover in the context of proteostasis [1,2,5,8,10,11] and cellular responses to stress, when assembled into stress granules [18]. However, in the absence of stress, TIA proteins have been involved in promoting polysome association [73]. In this regard, recent observations have suggested that the presence of these multifunctional proteins could increase protein expression in subcellular compartments, but especially in the context of the endoplasmic reticulum with a cooperative effect on translation [73]. However, this cellular context-dependent behavior could be connected to additional currently unknown components. But perhaps the most relevant of these observations is to point out the functional duality of these master regulators that as a consequence of a change in the localization of protein synthesis within the cytoplasm strongly influences protein production, indicating that a change in TIA1/TIAR-dependent mRNA localization could modify the relative abundance of certain specific proteins [73]. These findings expand and reinforce the regulatory capacity of these multifunctional proteins as molecular sensors of cellular and environmental needs in homeostasis and under stress situations, respectively. In the same line, the dual role of TIA1/TIAR proteins as tumor suppressors and enhancers in a cell/tissue type-dependent way in tumorigenesis suggest overlapping, cooperative and/or antagonistic regulatory roles between its protein variants [23,34,35,36,37,38] and the complexity of the combinatorial dynamic nature of RNA–RNA and RNA–protein interactome at the crossroads with heterogeneous populations of protein non-coding and/or encoding RNAs and other additional RBPs [4,6,74]. Further, TIA1 and TIAR are two abundant proteins in many eukaryotic cells. Thus, a recent study estimated its concentrations in HEK-293T cells at around 630 nM and 3.8 × 105 copies/cell and 1100 nM and 6.9 × 105 copies/cell, respectively [75].

Overall, the co-occurrence network analysis highlighted the multifaceted involvement of TIA1 in lymphoma biology and immunology, its potential as a valuable tool for understanding disease mechanisms and patient outcomes, its central role in orchestrating the cytotoxic functions of T-lymphocytes and its relevance as a potential immunological biomarker. Likewise, the co-occurrence network of TIA1 reflected its versatility and importance in fundamental cellular processes and disease mechanisms. Moreover, the TIAR co-occurrence network reflects the multifaceted nature of TIAR research, highlighting its importance in RNA biology, protein biosynthesis and the molecular mechanisms underlying gene expression regulation.

As we mentioned above, the GO analysis revealed results in two areas: the molecular function and functional processes of TIA1 and TIAR, and their implication in disease. The biological processes and molecular functions in which both proteins are involved are quite similar and relate to the regulation of gene expression. However, the difference in the RNAs they interact with is reflected in the diseases that they are likely to be involved in, with TIA1 being involved in neuronal diseases and TIAR being associated more with types of cancer.

To sum up, this bibliometric analysis of TIA1 and TIAR proteins shows that both are well represented in various disciplines, including molecular biology, biochemistry, cell biology and immunology. Notably, the volume of research conducted on these proteins has been steadily increasing, indicating a growing interest in this research area. The GO analysis, which includes the gene sets with which TIA1 and TIAR interact, provides a good idea of the pathologies in which they could be indirectly involved. Indeed, TIA1 and TIAR are likely to be involved upstream in pathologies with which they have not previously been implicated.

## 7. Conclusions

In this review, we have focused on bibliometric data related to TIA1/TIA-1 and TIAL1/TIAR genes/proteins and their quantitative and qualitative overviews on molecular, cellular and pathophysiological aspects. Although in recent years there have been great and relevant advances in this regard, there are still many questions that remain to be answered and that deserve more detailed study. For instance, the differential aspects related to regulatory adaptations and cellular dynamics among transcriptome–transcriptome–translatome–proteome and interactomes associated with each of the main cell-, tissue- and species-expressing TIA1 and TIAR variants in homeostasis, stress and pathological situations remain to be investigated and established. Moreover, since current technology allows it, obtaining single-cell transcriptomic and proteomic expression profiling could provide novel and much more precise information concerning the role of TIA proteins in different cellular types, developmental stages and many aging-associated pathologies. Furthermore, the ultimate and perhaps the most important challenge to undertake should be the identification of prognostic, diagnostic and/or predictive targets together with the development of therapeutic strategies in improving the functional and organismal roles exerted by these master regulatory biomarkers and gatekeepers. This research opens the door to future consideration of these and other RBPs when investigating the origins or interactions of a wide range of human aging-related pathologies.

## Figures and Tables

**Table 1:** Top 10 journals of publications on TIA1/TIA-1 and TIAR/TIAL1, respectively.

Protein	Journals	Country	Documents	IF
TIA1	Zhonghua Bing Li Xue Za Zhi = Chinese Journal of Pathology	China	38	8.0
	The American Journal of Surgical Pathology	USA	35	6.298
	Histopathology	UK	24	7.778
	Journal of Virology	USA	24	6.549
	PLoS One	USA	24	3.752
	Journal of Cutaneous Pathology	Denmark	21	1.458
	Molecular and Cellular Biology	USA	21	1.68
	Virchows Archive: An International Journal of Pathology	Germany	20	1.709
	The American Journal of Dermatopathology	USA	19	1.391
	Pathology	USA	18	3.526
TIAR	The Journal of Biological Chemistry	USA	17	5.486
	Molecular and Cellular Biology	USA	14	5.094
	Journal of Virology	USA	12	10.1
	PLoS One	USA	10	3.752
	Nucleic Acids Research	UK	9	19.160
	Biochemical and Biophysical Research Communications	USA	6	3.575
	International Journal of Molecular Sciences	Switzerland	5	6.208
	Molecular Biology of the Cell	USA	5	3.612
	Molecular Cell	USA	5	19.328
	Proceedings of the National Academy of Sciences (USA)	USA	5	12.779

## Data Availability

Not applicable.

## References

[1] Tian Q., Streuli M., Saito H., Schlossman S.F., Anderson P. (1991). A polyadenylate binding protein localized to the granules of cytolytic lymphocytes induces DNA fragmentation in target cells. Cell.

[2] Kawakami A., Tian Q., Streuli M., Poe M., Edelhoff S., Disteche C.M., Anderson P. (1994). Intron-exon organization and chromosomal localization of the human TIA-1 gene. J. Immunol. Baltim. Md. 1950.

[3] Izquierdo J.M., Valcárcel J. (2007). Two isoforms of the t-cell intracellular antigen 1 (TIA-1) splicing factor display distinct splicing regulation activities: Control of TIA-1 isoform ratio by TIA-1-related protein. J. Biol. Chem..

[4] Fernández-Gómez A., Izquierdo J.M. (2022). The multifunctional faces of T-cell intracellular antigen 1 in health and disease. Int. J. Mol. Sci..

[5] Kawakami A., Tian Q., Duan X., Streuli M., Schlossman S.F., Anderson P. (1992). Identification and functional characterization of a TIA-1-related nucleolysin. Proc. Natl. Acad. Sci. USA.

[6] Velasco B.R., Izquierdo J.M. (2022). T-cell intracellular antigen 1-like protein in physiology and pathology. Int. J. Mol. Sci..

[7] Aznarez I., Barash Y., Shai O., He D., Zielenski J., Tsui L.-C., Parkinson J., Frey B.J., Rommens J.M., Blencowe B.J. (2008). A systematic analysis of intronic sequences downstream of 5′ splice sites reveals a widespread role for U-rich motifs and TIA1/TIAL1 proteins in alternative splicing regulation. Genome Res..

[8] Dember L.M., Kim N.D., Liu K.Q., Anderson P. (1996). Individual RNA recognition motifs of TIA-1 and TIAR have different RNA binding specificities. J. Biol. Chem..

[9] Meyer C., Garzia A., Mazzola M., Gerstberger S., Molina H., Tuschl T. (2018). The TIA1 RNA-binding-protein family regulates EIF2AK2-mediated stress response and cell cycle progression. Mol. Cell.

[10] López de Silanes I., Galbán S., Martindale J.L., Yang X., Mazan-Mamczarz K., Indig F.E., Falco G., Zhan M., Gorospe M. (2005). Identification and functional outcome of mRNAs associated with RNA-binding protein TIA-1. Mol. Cell Biol..

[11] Mazan-Mamczarz K., Lal A., Martindale J.L., Kawai T., Gorospe M. (2006). Translational repression by RNA-binding protein TIAR. Mol. Cell Biol..

[12] Sidali A., Teotia V., Solaiman N.S., Bashir N., Kanagaraj R., Murphy J.J., Surendranath K. (2021). AU-rich element RNA binding proteins: At the crossroads of post-transcriptional regulation and genome integrity. Int. J. Mol. Sci..

[13] Barron V.A., Lou H. (2011). Alternative splicing of the neurofibromatosis type I pre-mRNA. Biosci. Rep..

[14] Wang Z., Kayikci M., Briese M., Zarnack K., Luscombe N.M., Rot G., Zupan B., Curk T., Ule J. (2010). iCLIP Predicts the dual splicing effects of TIA-RNA interactions. PLoS Biol..

[15] Zhao W., Zhao J., Hou M., Wang Y., Zhang Y., Zhao X., Zhang C., Guo D. (2014). HuR and TIA1/TIAL1 are involved in regulation of alternative splicing of SIRT1 pre-mRNA. Int. J. Mol. Sci..

[16] Anderson P., Kedersha N. (2002). Stressful initiations. J. Cell Sci..

[17] Jain S., Wheeler J.R., Walters R.W., Agrawal A., Barsic A., Parker R. (2016). ATPase-modulated stress granules contain a diverse proteome and substructure. Cell.

[18] Kedersha N.L., Gupta M., Li W., Miller I., Anderson P. (1999). RNA-binding proteins TIA-1 and TIAR link the phosphorylation of eIF-2α to the assembly of mammalian stress granules. J. Cell Biol..

[19] Waris S., Wilce M.C.J., Wilce J.A. (2014). RNA recognition and stress granule formation by TIA proteins. Int. J. Mol. Sci..

[20] Arimoto-Matsuzaki K., Saito H., Takekawa M. (2016). TIA1 Oxidation inhibits stress granule assembly and sensitizes cells to stress-induced apoptosis. Nat. Commun..

[21] Bossowski A., Czarnocka B., Bardadin K., Moniuszko A., Łyczkowska A., Czerwinska J., Dadan J., Bossowska A. (2010). Identification of chosen apoptotic (TIAR and TIA-1) markers expression in thyroid tissues from adolescents with immune and non-immune thyroid diseases. Folia Histochem. Cytobiol..

[22] Förch P., Puig O., Kedersha N., Martínez C., Granneman S., Séraphin B., Anderson P., Valcárcel J. (2000). The apoptosis-promoting factor TIA-1 is a regulator of alternative pre-mRNA splicing. Mol. Cell.

[23] Reyes R., Alcalde J., Izquierdo J.M. (2009). Depletion of T-cell intracellular antigen proteins promotes cell proliferation. Genome Biol..

[24] Beck A.R.P., Miller I.J., Anderson P., Streuli M. (1998). RNA-binding protein TIAR is essential for primordial germ cell development. Proc. Natl. Acad. Sci. USA.

[25] Sánchez-Jiménez C., Izquierdo J.M. (2013). T-cell intracellular antigen (TIA)-proteins deficiency in murine embryonic fibroblasts alters cell cycle progression and induces autophagy. PLoS ONE.

[26] Geng Z., Li P., Tan L., Song H. (2015). Targeted knockdown of RNA-binding protein TIAR for promoting self-renewal and attenuating differentiation of mouse embryonic stem cells. Stem Cells Int..

[27] Scheu S., Stetson D.B., Reinhardt R.L., Leber J.H., Mohrs M., Locksley R.M. (2006). Activation of the integrated stress response during T helper cell differentiation. Nat. Immunol..

[28] Barron V.A., Zhu H., Hinman M.N., Ladd A.N., Lou H. (2010). The neurofibromatosis type I pre-mRNA is a novel target of CELF protein-mediated splicing regulation. Nucleic Acids Res..

[29] Rayman J.B., Hijazi J., Li X., Kedersha N., Anderson P.J., Kandel E.R. (2019). Genetic Perturbation of TIA1 reveals a physiological role in fear memory. Cell Rep..

[30] Vanderweyde T., Youmans K., Liu-Yesucevitz L., Wolozin B. (2013). Role of stress granules and RNA-binding proteins in neurodegeneration: A mini-review. Gerontology.

[31] Vanderweyde T., Yu H., Varnum M., Liu-Yesucevitz L., Citro A., Ikezu T., Duff K., Wolozin B. (2012). Contrasting pathology of the stress granule proteins TIA-1 and G3BP in tauopathies. J. Neurosci..

[32] Waelter S., Boeddrich A., Lurz R., Scherzinger E., Lueder G., Lehrach H., Wanker E.E. (2001). Accumulation of mutant huntingtin fragments in aggresome-like inclusion bodies as a result of insufficient protein degradation. Mol. Biol. Cell.

[33] Yuan Z., Jiao B., Hou L., Xiao T., Liu X., Wang J., Xu J., Zhou L., Yan X., Tang B. (2018). Mutation analysis of the TIA1 gene in chinese patients with amyotrophic lateral sclerosis and frontotemporal dementia. Neurobiol. Aging..

[34] Izquierdo J.M., Alcalde J., Carrascoso I., Reyes R., Ludeña M.D. (2011). Knockdown of T-cell intracellular antigens triggers cell proliferation, invasion and tumour growth. Biochem. J..

[35] Liu Y., Liu R., Yang F., Cheng R., Chen X., Cui S., Gu Y., Sun W., You C., Liu Z. (2017). miR-19a Promotes colorectal cancer proliferation and migration by targeting TIA1. Mol. Cancer.

[36] Sánchez-Jiménez C., Ludeña M.D., Izquierdo J.M. (2015). T-cell intracellular antigens function as tumor suppressor genes. Cell Death Dis..

[37] Yang X., Wang M., Lin B., Yao D., Li J., Tang X., Li S., Liu Y., Xie R., Yu S. (2018). miR-487a promotes progression of gastric cancer by targeting TIA1. Biochimie.

[38] Liu J., Cao X. (2023). RBP–RNA interactions in the control of autoimmunity and autoinflammation. Cell Res..

[39] Naz S., Khan R.A., Giddaluru J., Battu S., Vishwakarma S.K., Subahan M., Satti V., Khan N., Khan A.A. (2018). Transcriptome meta-analysis identifies immune signature comprising of RNA binding proteins in ulcerative colitis patients. Cell Immunol..

[40] Dinh P.X., Beura L.K., Das P.B., Panda D., Das A., Pattnaik A.K. (2013). Induction of stress granule-like structures in vesicular stomatitis virus-infected cells. J. Virol..

[41] Le Sage V., Cinti A., McCarthy S., Amorim R., Rao S., Daino G.L., Tramontano E., Branch D.R., Mouland A.J. (2017). Ebola virus VP35 blocks stress granule assembly. Virology.

[42] Lloyd R.E. (2015). Nuclear proteins hijacked by mammalian cytoplasmic plus strand RNA viruses. Virology.

[43] McCormick C., Khaperskyy D.A. (2017). Translation inhibition and stress granules in the antiviral immune response. Nat. Rev. Immunol..

[44] Sun Y., Dong L., Yu S., Wang X., Zheng H., Zhang P., Meng C., Zhan Y., Tan L., Song C. (2017). Newcastle disease virus induces stable formation of bona fide stress granules to facilitate viral replication through manipulating host protein translation. FASEB J..

[45] LeBlang C.J., Medalla M., Nicoletti N.W., Hays E.C., Zhao J., Shattuck J., Cruz A.L., Wolozin B., Luebke J.I. (2020). Reduction of the RNA binding protein TIA1 exacerbates neuroinflammation in tauopathy. Front. Neurosci..

[46] Piecyk M., Wax S., Beck A.R., Kedersha N., Gupta M., Maritim B., Chen S., Gueydan C., Kruys V., Streuli M. (2000). TIA-1 is a translational silencer that selectively regulates the expression of TNF-alpha. EMBO J..

[47] Simarro M., Giannattasio G., Xing W., Lundequist E.-M., Stewart S., Stevens R.L., Orduña A., Boyce J.A., Anderson P.J. (2012). The translational repressor T-cell intracellular antigen-1 (TIA-1) is a key modulator of Th2 and Th17 responses driving pulmonary inflammation induced by exposure to house dust mite. Immunol. Lett..

[48] Yu C., York B., Wang S., Feng Q., Xu J., O’Malley B.W. (2007). An essential function of the SRC-3 coactivator in suppression of cytokine mRNA translation and inflammatory response. Mol. Cell.

[49] Aria M., Cuccurullo C. (2017). Bibliometrix: An R-tool for comprehensive science mapping analysis. J. Informetr..

[50] Maziuk B., Ballance H.I., Wolozin B. (2017). Dysregulation of RNA binding protein aggregation in neurodegenerative disorders. Front. Mol. Neurosci..

[51] Nguyen T.T., Frater J.L., Klein J., Chen L., Bartlett N.L., Foyil K.V., Kreisel F.H. (2016). Expression of TIA1 and PAX5 in classical hodgkin lymphoma at initial diagnosis may predict clinical outcome. Appl. Immunohistochem. Mol. Morphol. AIMM.

[52] Hong W., Hu Y., Fan Z., Gao R., Yang R., Bi J., Hou J. (2020). In silico identification of EP400 and TIA1 as critical transcription factors involved in human hepatocellular carcinoma relapse. Oncol. Lett..

[53] Hamada J., Shoda K., Masuda K., Fujita Y., Naruto T., Kohmoto T., Miyakami Y., Watanabe M., Kudo Y., Fujiwara H. (2016). Tumor-promoting function and prognostic significance of the RNA-binding protein T-cell intracellular antigen-1 in esophageal squamous cell carcinoma. Oncotarget.

[54] Ren X., Liu W., Li G., Li F., Zhang S. (1999). Epstein-Barr virus infection and expression of T-cell intracellular antigen-1 (TIA-1) in intestinal T-cell lymphoma. Zhonghua Bing. Li Xue Za Zhi.

[55] Zlobec I., Karamitopoulou E., Terracciano L., Piscuoglio S., Iezzi G., Muraro M.G., Spagnoli G., Baker K., Tzankov A., Lugli A. (2010). TIA-1 Cytotoxic granule-associated RNA binding protein improves the prognostic performance of CD8 in mismatch repair-proficient colorectal cancer. PLoS ONE.

[56] Mori N., Murakami Y.I., Shimada S., Iwamizu-Watanabe S., Yamashita Y., Hasegawa Y., Kojima H., Nagasawa T. (2004). TIA-1 expression in hairy cell leukemia. Mod. Pathol..

[57] Boulland M.L., Kanavaros P., Wechsler J., Casiraghi O., Gaulard P. (1997). Cytotoxic protein expression in natural killer cell lymphomas and in alpha beta and gamma delta peripheral T-cell lymphomas. J. Pathol..

[58] Kim H.S., Kuwano Y., Zhan M., Pullmann R., Mazan-Mamczarz K., Li H., Kedersha N., Anderson P., Wilce M.C.J., Gorospe M. (2007). Elucidation of a C-rich signature motif in target mRNAs of RNA-binding protein TIAR. Mol. Cell Biol..

[59] Taupin J.L., Tian Q., Kedersha N., Robertson M., Anderson P. (1995). The RNA-binding protein TIAR is translocated from the nucleus to the cytoplasm during Fas-mediated apoptotic cell death. Proc. Natl. Acad. Sci. USA.

[60] Kedersha N., Cho M.R., Li W., Yacono P.W., Chen S., Gilks N., Golan D.E., Anderson P. (2000). Dynamic shuttling of TIA-1 accompanies the recruitment of mRNA to mammalian stress granules. J. Cell Biol..

[61] Kedersha N., Chen S., Gilks N., Li W., Miller I.J., Stahl J., Anderson P. (2002). Evidence that ternary complex (eIF2-GTP-tRNA(i)(Met))-deficient preinitiation complexes are core constituents of mammalian stress granules. Mol. Biol. Cell.

[62] Dean J.L.E., Sully G., Clark A.R., Saklatvala J. (2004). The involvement of AU-rich element-binding proteins in p38 mitogen-activated protein kinase pathway-mediated mRNA stabilisation. Cell Signal..

[63] Duttagupta R., Tian B., Wilusz C.J., Khounh D.T., Soteropoulos P., Ouyang M., Dougherty J.P., Peltz S.W. (2005). Global analysis of pub1p targets reveals a coordinate control of gene expression through modulation of binding and stability. Mol. Cell Biol..

[64] García-Mauriño S.M., Rivero-Rodríguez F., Velázquez-Cruz A., Hernández-Vellisca M., Díaz-Quintana A., De la Rosa M.A., Díaz-Moreno I. (2017). RNA binding protein regulation and cross-talk in the control of AU-rich mRNA fate. Front. Mol. Biosci..

[65] Stoecklin G., Stubbs T., Kedersha N., Wax S., Rigby W.F.C., Blackwell T.K., Anderson P. (2004). MK2-induced Tristetraprolin:14-3-3 complexes prevent stress granule association and ARE-mRNA decay. EMBO J..

[66] Katsanou V., Papadaki O., Milatos S., Blackshear P.J., Anderson P., Kollias G., Kontoyiannis D.L. (2005). HuR as a negative posttranscriptional modulator in inflammation. Mol. Cell.

[67] Akira S., Maeda K. (2021). Control of RNA stability in immunity. Annu. Rev. Immunol..

[68] van Eck N.J., Waltman L., Ding Y., Rousseau R., Wolfram D. (2014). Visualizing bibliometric networks. Measuring Scholarly Impact: Methods and Practice.

[69] Carrascoso I., Alcalde J., Tabas-Madrid D., Oliveros J.C., Izquierdo J.M. (2018). transcriptome-wide analysis links the short-term expression of the b isoforms of TIA proteins to protective proteostasis-mediated cell quiescence response. PLoS ONE.

[70] Chen E.Y., Tan C.M., Kou Y., Duan Q., Wang Z., Meirelles G.V., Clark N.R., Ma’ayan A. (2013). Enrichr: Interactive and collaborative HTML5 gene list enrichment analysis Tool. BMC Bioinform..

[71] Kuleshov M.V., Jones M.R., Rouillard A.D., Fernandez N.F., Duan Q., Wang Z., Koplev S., Jenkins S.L., Jagodnik K.M., Lachmann A. (2016). Enrichr: A comprehensive gene set enrichment analysis web server 2016 update. Nucleic Acids Res..

[72] Xie Z., Bailey A., Kuleshov M.V., Clarke D.J.B., Evangelista J.E., Jenkins S.L., Lachmann A., Wojciechowicz M.L., Kropiwnicki E., Jagodnik K.M. (2021). Gene set knowledge discovery with Enrichr. Curr. Protoc..

[73] Horste E.L., Fansler M.M., Cai T., Chen X., Mitschka S., Zhen G., Lee F.C.Y., Ule J., Mayr C. (2023). Subcytoplasmic location of translation controls protein output. Mol. Cell.

[74] Van Nostrand E.L., Freese P., Pratt G.A., Wang X., Wei X., Xiao R., Blue S.M., Chen J.Y., Cody N.A.L., Dominguez D. (2020). A large-scale binding and functional map of human RNA-binding proteins. Nature.

[75] Cho N.H., Cheveralls K.C., Brunner A.D., Kim K., Michaelis A.C., Raghavan P., Kobayashi H., Savy L., Li J.Y., Canaj H. (2022). OpenCell: Endogenous tagging for the cartography of human cellular organization. Science.

